# Draft Genome Sequences of Three *Capnocytophaga canimorsus* Strains Isolated from Septic Patients

**DOI:** 10.1128/genomeA.00193-15

**Published:** 2015-05-28

**Authors:** Pablo Manfredi, Francesco Renzi, Guy R. Cornelis

**Affiliations:** a Biozentrum der Universität Basel, Basel, Switzerland; b University of Namur, Namur, Belgium

## Abstract

*Capnocytophaga canimorsus* is a bacterium from the normal oral flora of dogs and cats that causes rare generalized infections in humans. In an attempt to determine whether infections could be caused by a subset of strains and to identify pathogenicity factors, we sequenced the genomes of three strains isolated from human infections.

## GENOME ANNOUNCEMENT

*Capnocytophaga canimorsus* is a Gram-negative commensal bacterium from the normal canine oral flora that causes life-threatening septicemia in patients who have been in contact with dogs or cats (1). Infections present with fulminant sepsis (2, 3), including cases of meningitis, endocarditis, or myocarditis. Fastidious growth of the pathogen and lack of symptoms during the initial stages of infection often lead to an unattended wound (4), resulting in a mortality rate of as high as 30% (1). Predisposing factors, like splenectomy (33%) or alcohol abuse (24%), have indeed been reported, but in 41% of the cases, patients do not show any obvious risk factors (1). Recent molecular studies on the pathogenicity of the strain Cc5 (5) led to the identification of different pathogenic factors of *C. canimorsus* (6–12). In particular, the data presented here allowed the identification of a new type of iron import system required for growth of the pathogen in human serum (13).

The three clinical strains of *C. canimorsus*, Cc2, Cc11, and Cc12 (i.e., ATCC 35979), were isolated from human patients (blood samples) who developed septicemia (7). Genomic DNA was extracted using the Genomic-tip 500/G DNA extraction kit (catalog no. 10262; Qiagen), according to the manufacturer’s instructions, followed by an additional phenol-chloroform purification step. A total of 1.3 million (Cc11), 2.3 million (Cc12), and 2.4 million (Cc2) paired-end microreads (36 bp; fragment length, 205 ± 50 bp) were generated at Fasteris SA, Geneva (Switzerland), from a single run of Solexa/Illumina GAII EAS269 on 100 tiles. Additional sequencing data were generated with the Roche genome sequencing FLX system DNA pyrosequencing at Microsynth, Balgach, Switzerland, corresponding to 75,000 to 80,000 reads per strain of approximately 315-bp read length. The assemblies also included primer walking Sanger sequencing data generated on cherry-picked regions. The final hybrid assembly was performed with MIRA (14) and included pseudoreads corresponding to contigs mapped onto the reference chromosome of *C. canimorsus* strain 5 (Cc5) (5) using MAQ (15) and to contigs from the *de novo* assembly generated with Velvet with optimized parameters (16). Genome annotation and preliminary analyses were performed by LABGeM, France Génomique (17). The genomic metrics of the three draft assemblies (206 [Cc2], 249 [Cc11], and 81 [Cc12] contigs) were similar to those of Cc5 (5), with draft assembly sizes ranging from 2.39 to 2.52 Mb (2.57 for Cc5), G+C content between 36.08% and 36.23% (36.11% for Cc5), and a total of 2,702 to 2,874 coding sequences (CDS) detected (2,519 for Cc5). The *C. canimorsus* core genome based on the four strains mentioned above included 1,292 genes, which corresponded to only 45 to 51% of the total genes in each strain, and therefore indicates high genomic plasticity within the taxon. With respect to pathogenicity factor candidates, 177 clusters of orthologs were found conserved in the four clinical isolates but not in six additional *C. canimorsus* strains isolated from a dog’s mouth (18). Also, 118 clusters with unknown function, 18 genes involved in oxidative respiration, 10 in ion and peptide transport, 10 in mobile element transposition, 4 in transcriptional regulation, and 3 in cell adhesion formed the predominant functional classes of the list.

### Nucleotide sequence accession numbers. 

These whole-genome shotgun projects have been deposited in ENA under the accession numbers CDOJ00000000 (Cc2), CDOK00000000 (Cc11), and CDOE00000000 (Cc12). The versions described in this paper are the initial versions.
